# Study on the effect of synergy effect between the mixed cultures on the power generation of microbial fuel cells

**DOI:** 10.1080/21655979.2021.1883280

**Published:** 2021-03-08

**Authors:** Jing Ren, Na Li, Maohua Du, Yixin Zhang, Chunxu Hao, Rui Hu

**Affiliations:** a School of Environment, Liaoning University, Shenyang, Liaoning, China; b Environment Planning Institute, Ministry of Ecology and Environment, Beijing, China

**Keywords:** Microbial fuel cells (MFC), the mixed cultures, synergy effect, the power generation, exogenous electron mediator

## Abstract

Microbial fuel cells (MFC) can use microorganisms to directly convert the chemical energy of organic matter into electrical energy, and generate electrical energy while pollutants degradation. To solve the critical problem of lower power yield of power production, this study selected *Saccharomyces cerevisiae, Escherichia coli, Pseudomonas aeruginosa,* and *Bacillus subtilis* as the anodic inoculums. The influence of the mixed bacteria on the power-producing effect of MFC and the synergy effect between the electrochemically active bacteria in mixed cultures were discussed. The results showed that among the mixed culture system, only the mixed cultures MFC composed of *Saccharomyces cerevisiae* and *Bacillus subtilis* had a significant increase in power generation capacity, which could reach to 554 mV. Further analysis of the electrochemical and microbiological performance of this system was conducted afterward to verify the synergy effect between *Saccharomyces cerevisiae* and *Bacillus subtilis*. The riboflavin produced by *Bacillus subtilis* could be utilized by *Saccharomyces cerevisiae* to enhance the power generation capacity. Meanwhile, *Saccharomyces cerevisiae* could provide carbon source and electron donor for *Bacillus subtilis* through respiration. Finally, in the experiment of adding exogenous riboflavin in the mixed bacterial MFC, the result indicated that the mixed bacterial MFC chose the self-secreting riboflavin over the exogenous riboflavin as the electron mediator, and the excess riboflavin might hinder the electron trasfer.

## Graphical abstract

**Figure:**
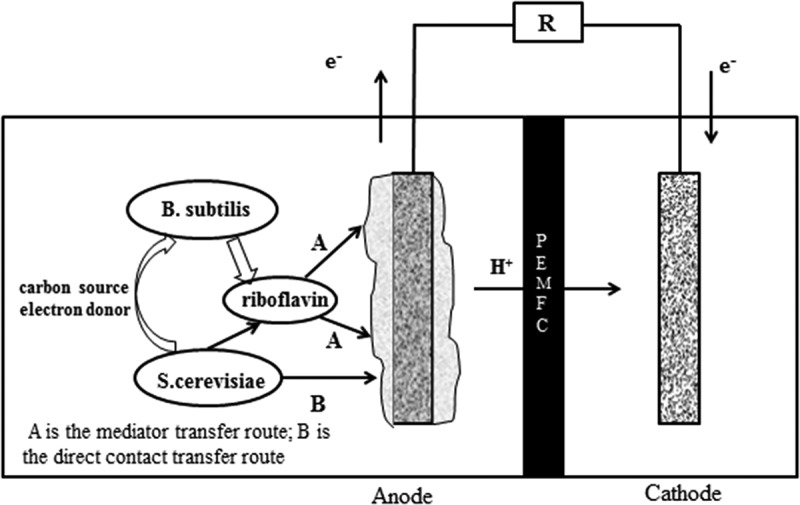

## 1. Introduction

As over-exploited of fossil fuels and other nonrenewable energy resources, issues such as environmental pollution and energy shortage become more prominent. In this context, developing renewable energy as an alternative is thus a pressing task for us. Microbial fuel cells (MFC) can generate electricity during treating sewage, electrochemically active bacteria in the MFC can translate the chemical energy in simple small molecules or complex biomass into electrical energy or hydrogen energy directly [1]. MFC has the advantages of diversified fuel sources, mild operating conditions, no pollution or energy input, and high-efficiency energy utilization [2,3]. However, lower power yield has always been a major factor restricting MFC from theory to practical application.

There are three main mechanisms for the electrons transfer to the anode by bacteria in the MFC anode chamber: direct contact transfer [4], nanowire [5], and electron shuttle transfer [6]. Direct contact refers to the transfer of electrons produced by electrically active bacteria from microbial cells to electrodes through cytochromes [7]. Nanowire transfer mechanism could be achieved by fimbriae on the cell membrane of electric microorganisms, the fimbriae could help electron transfer by connecting with an electrode [8]. Studies have shown that the output voltage is related to the number of microorganisms on the surface of the electrode per unit area [9]. Some microorganisms in MFC can metabolize to produce electronic shuttles (riboflavin [10], Phenazine [11], etc.) served as the electron mediator. Besides, an external electron mediator such as neutral red [12], can also be artificially added to the MFC for electron transfer.

At present, great progress has been made in the research on the power production mechanism of the pure cultures, but there are certain difficulties in maintaining the pure cultures [13]. Some studies have shown that proper mixed bacterial inoculation can achieve the same pollutant treatment effect as pure bacterial inoculation [14], while the power generated by the mixed cultures MFC is higher than that of the pure cultures MFC [15]. This is because the synergy effect among electrochemically active bacterias also plays a key role in efficient electron transfer when mixed cultures were used during MFC operation [16]. Cao et al. [17] constructed a mixed culture system of *Sulfur-reducing bacteria* and *E. coli* and compared it with purely cultured *Sulfur-reducing bacteria*. It is found that the power generation capacity and the ability to adapt to complex environments of the pure cultures MFC are lower than those of the mixed cultures MFC. Islam et al. [18] found that the synergy effect between *Pseudomonas aeruginosa* and *Klebsiella acne* could produce a more efficient electronic shuttle medium to enhance the power generation, and the maximum current density obtained was about three times as much as that of pure culture. However, not every mixed culture could produce a significant effect on power production because of the symbiotic, cooperative, and antagonistic relationships among the microorganisms [19]. And after inoculation with the mixed cultures, it is difficult to determine the electron transfer mechanism between microorganisms. Understanding the interaction between microorganisms and the metabolic network in the mixed culture system is of great significance for improving the power generation capacity of MFC.

To increase the output power of MFC, clarify the influence of mixed culture on the power generation performance of MFC, this study constructed mixed cultures MFC systems including *Saccharomyces cerevisiae* [20], *Escherichia coli* [21], *Pseudomonas aeruginosa* [22], and *Bacillus subtilis* [23] as the anodic inoculums, based on different electron transfer mechanisms from the microorganism to the electrode. Study the influence of the interaction between microorganisms on MFC power generation. Then use cyclic voltammetry, polarization curve, and biofilm analysis to further explore the mechanism between mixed culture MFC anode microorganisms, in order to provide a theoretical basis for the theoretical study of mixed bacteria MFC.

## 2. Materials and methods

### 2.1. Experimental materials and instruments

#### 2.1.1. Selection and cultivation of strains

Four electrochemically active bacteria are studied and used to construct mixed bacteria based on their features: *Saccharomyces cerevisiae, Escherichia coli, Bacillus subtilis, Pseudomonas Aeruginosa*. All bacteria were purchased from Huankai Biologics. Among them, *Saccharomyces cerevisiae* took potato extract glucose medium, and the other three took the beef extract-peptone medium to foster the bacteria. Autoclaved these four culture media for 20 min and adjusted the pH to 7.4–7.6.

#### 2.1.2. Experimental setup

A dual-chamber MFC device was chosen in the experiments, the volume of each chamber was 125 mL. The treated carbon cloth (2 cm×2 cm) was used as the cathode and anode of the battery, respectively. The anode and cathode chambers, separated by proton exchange membrane (effective area of 16 cm^2^), are equipped with a sampling hole and aeration hole with sealing plugs on their respective top, with an external resistance of 1000 Ω.

Anolyte was composed of 1 g/L glucose solution, 50 mM phosphate buffer, mineral solution, and vitamin solution. The catholyte was made up of 32.925 g of potassium ferricyanide, 0.9331 g of Na_2_HPO_4_﹒12H_2_O, 4.77 g of NaH_2_PO_4_﹒2H_2_O, and adjusted the pH of the two solutions to 7.0.

### 2.2. Experimental methods

#### 2.2.1. Electrochemical testing

The performance of MFC was evaluated by measuring open-circuit voltage (OCV), cyclic voltammetry (CV), polarization curve, and power density curve of MFC. Set the external circuit load to 1000 Ω by a digital multimeter, and measured the output voltage of the MFC. Used CV for electrochemical analysis of the mixed cultures, established the three electrodes by the electrochemical workstation (LK98BII). The scanning rate was 100 mV/s, and the scanning range was −0.1 V ~ 1 V, which can directly obtain the relevant CV curve. Finally, polarization curve and power density curve experiments were conducted to further analyzed the power generation performance of mixed cultures MFC. After the output voltage of the MFC system was stable, disconnected the circuit for 1 h, measured the open-circuit voltage value. Measured the voltage under different resistance values, sorted out the output voltage *U* under different external resistances.

The corresponding current *I* and current density *i* were calculated by Ohm’s law (equation 1 and 2), the power density *P* was calculated by equation 3. In which, *R* represented the resistance value and *A* was the electrode area.

(1) I=U/R1

(2) i=U/RA

(3) P=UI/A

The polarization curve was drawn by *U* versus *i*, and the power density curve was drawn by *P* versus *i* [24].

#### 2.2.2. Determination of microbiological properties

The morphology of the blank electrode and the electrode loaded with the mixed bacteria biofilm were analyzed by scanning electron microscope (SEM); the absorbance of the microorganisms was measured by the ultraviolet spectrophotometer (L5S) to obtain the concentration of the microorganism culture solution (OD600) to evaluate the electrochemically active bacteria the growth of the bacteria is judged by the cell density of the bacteria.

## 3. Results and discussion

### 3.1. Research on power generation performance

#### 3.1.1. Power generation performance of pure cultures MFC

**Figure 1.**
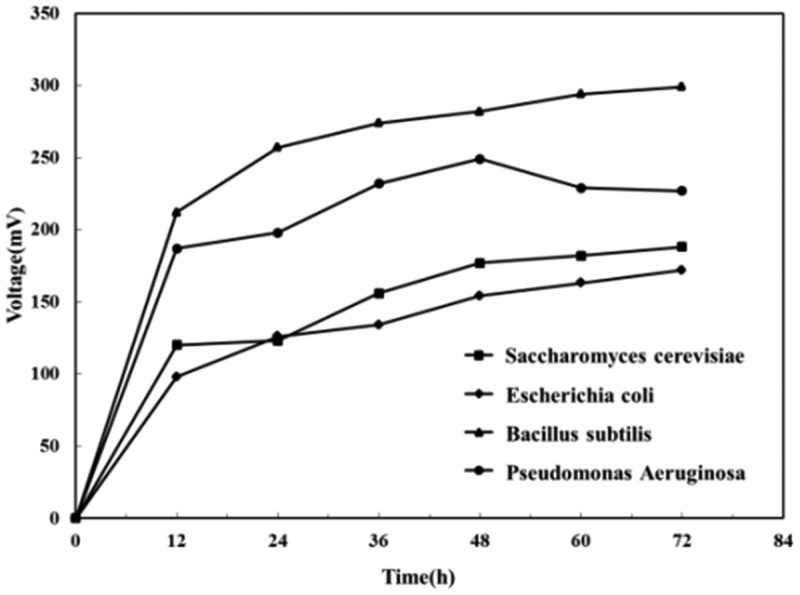
Power generation performance of pure cultures MFC

To investigated the power generation performance of each electrochemically active bacteria, the output voltage of MFC powered by each pure culture was measured at an external resistance of 1000 Ω. The output voltage of each electrochemically active bacteria over time was shown in Figure 1.

It could be seen from Figure 1 that the power generated by MFC with different electrochemically active bacteria increased over time, and each MFC showed a stable trend of output voltage after 60–72 h. Among the four MFCs, the *Bacillus subtilis* MFC outputed the highest voltage (229 mV), followed by the *Pseudomonas aeruginosa* MFC (227 mV), the *Saccharomyces cerevisiae* MFC (188 mV), and *E. coli* MFC (172 mV). Compared to the outputs of MFC with these microorganisms in the previous researches [25–28]. Our MFC indicated similar even better performance. It was worth noticing that, unliked other MFCs, the voltage of the *Pseudomonas aeruginosa* MFC suddenly dropped after rising to a peak, and then stabilizes. The result was consistent with the results of the study by Ting et al. [29]. The activity and survival of electron mediators and bacteria produced by *Pseudomonas aeruginosa* could directly affect the power production capacity of MFC, and the decrease in biofilm activity lowered the transfer rate between anode electrons and the anode over time, resulted in the drop of the output voltage.

#### 3.1.2. Power generation performance of electrochemically active bacteria in different combinations

**Figure 2.**
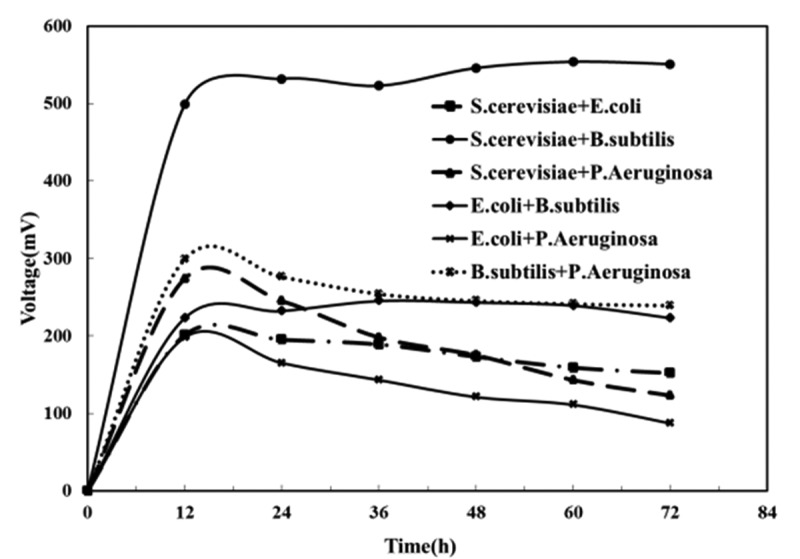
Output voltage of different mixed cultures MFC

We combined the above-mentioned electrochemically active bacteria as the anodic inoculums to construct mixed cultures MFC to investigate their power-producing performance. As shown in Figure 2, when each mixed cultures MFC outputed stable voltage, *Bacillus subtilis* and *Saccharomyces cerevisiae* mixed cultures MFC rose to the top in the output voltage, up to 554 mV, followed by *Bacillus subtilis* and *Pseudomonas aeruginosa* mixed cultures MFC (241 mV), *E. coli* and *Bacillus subtilis* (239 mV), *Saccharomyces cerevisiae* and *Escherichia coli* (159 mV), *Saccharomyces cerevisiae* and *Pseudomonas aeruginosa* (143 mV), *E. coli* and *P. aeruginosa* mixed cultures MFC (111 mV).

The MFC powered by different composite electrochemically active bacteria varied regarding power production performance. When *Saccharomyces cerevisiae* and *Escherichia coli* mixed with the other two, the former outperforms the latter in powering MFC to produce electricity as its stronger metabolic capacity made MFC powered by it faster in electron transfer despite the failure of both of them to produce intermediate mediators for electron transfer [10,30], shown by research. When *Bacillus subtilis* and *Pseudomonas aeruginosa* mixed with the other two, the former also outperforms the latter in powering MFC to produce electricity for the following two reasons: *Bacillus subtilis* is faster than *Pseudomonas aeruginosa* in electron transfer thanks to its stronger electrical activity, so it can reduce the internal resistance of MFC as the anode electrochemically active bacteria. Secondly, toxic chlorophyll [31] produced by *Pseudomonas aeruginosa* during metabolism may inhibit growth when coexisting with other microorganisms, making the anode biofilm less active. This leads to a decrease in output voltage.

### 3.2. Analysis of power production mechanism

The analysis on the power production performance of MFC powered by different combinations of electrochemically active bacteria indicated that no other combination performs better than *Bacillus subtilis* and *Saccharomyces cerevisiae* in this regard. In the following sections, the electricity production mechanism of MFC powered by the above-mixed bacteria is analyzed from the perspectives of electrochemistry and electron microscope scanning.

#### 3.2.1. CV analysis of mixed electrochemically active bacteria

**Figure 3.**
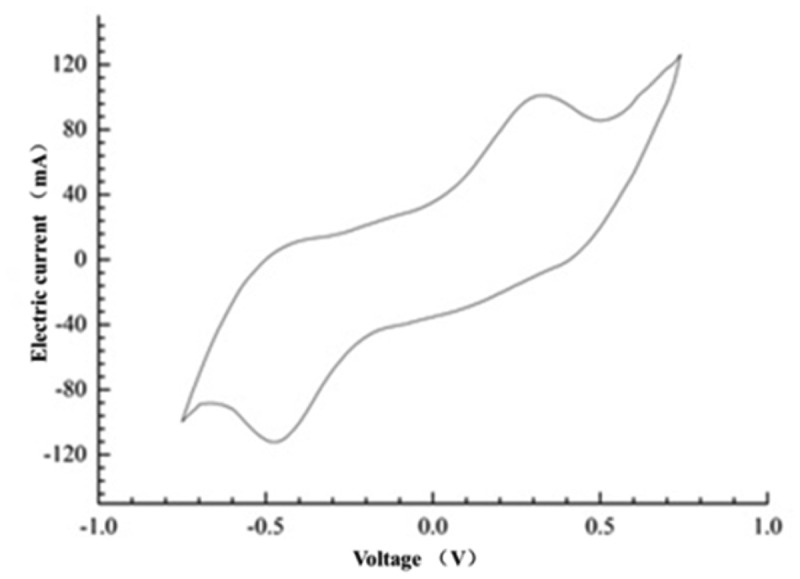
Cyclic voltammetry curve of mixed cultures MFC

It could be seen from the cyclic voltammetry curve in Figure 3 that a pair of relative redox peaked appear at 0.5 V and −0.5 V, which indicated that there might be free redox intermediates or membrane connecting substances attached to the anode biofilm of the mixed cultures MFC. The result was similar to the research result of Iama [18], which indicated that the electron transfer of the mixed culture MFC was completed by the oxidative substances secreted by the mixed bacteria or the membrane connecting substances of the electrochemically active bacteria. It can be inferred that the electron transfer process in the mixed cultures system might be the result of the cooperation of direct contact transfer and electron shuttle transfer mechanisms. The redox mediator secreted by *Bacillus subtilis* provided a channel for the transfer of anode electrons, thereby reducing the resistance in the process of electron transfer. As an endogenous electron mediator produced by electrochemically active bacteria, riboflavin played a very important role in electronic horizontal propagation [32,33].

#### 3.2.2. Polarization curve and power density curve of mixed cultures

**Figure 4.**
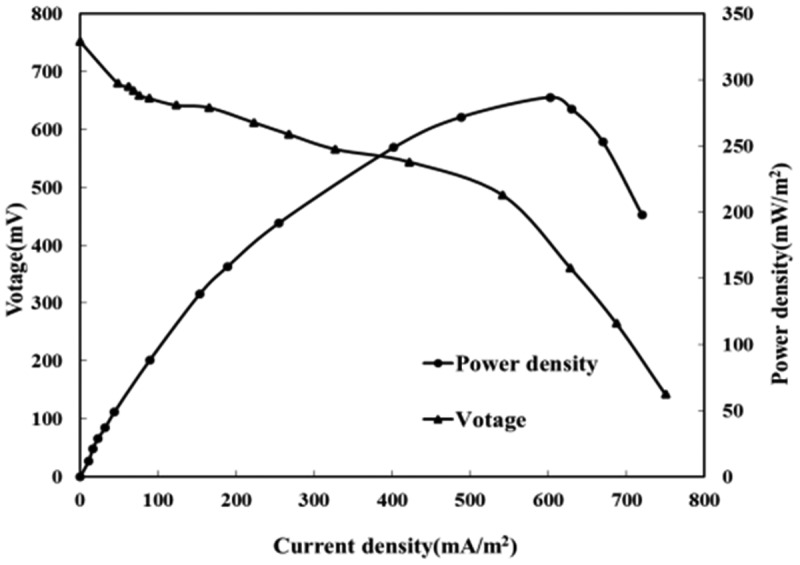
Polarization curve and power density curve of the compound strains

To better illustrate the power generation performance of the MFC used *Saccharomyces cerevisiae* and *Bacillus subtilis* as the mixed bacteria, the experiment measured the output voltage of MFC at different resistance values, calculated the current by the formula and delivered the power density curve and polarization curve as shown in Figure 4. The output voltage of the system is about 752 mV under an open-circuit, suggesting the high activity of electricity production. The power density curve shows that as the external resistance decreases, the current density and power density gradually increase. When the current reaches 602 mA/m^2^, the power density maximizes (287 mW/m^2^). After that, the power density begins to decline with the increasing current density.

#### 3.2.3. SEM analysis of anodic biomembrane

**Figure 5.**
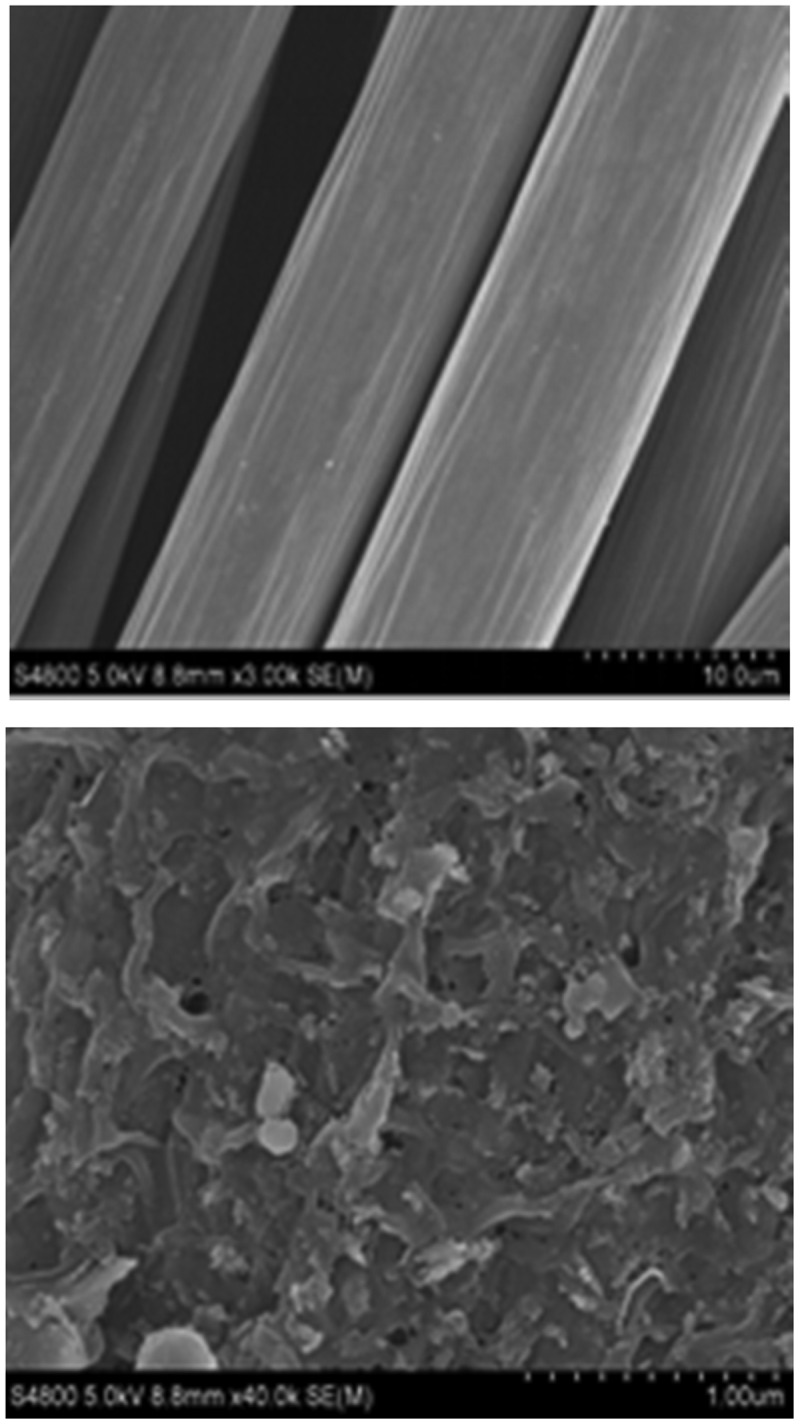
SEM photo of anodic biomembrane (a) blank electrode and (b) electrode of mixed bacteria

Figure 5(a) was a blank electrode without any electrochemically active bacteria load. It could be clearly seen that the surface of the carbon fiber is relatively smooth. Figure 5(b) was an electrode loaded with mixed cultured electrochemically active bacteria. It was not difficult to see that there were a large number of bacteria on the surface of the electrode. These electrochemically active bacterias interacted with the electrode surface to form a thick electrochemically active biofilm. Zhi et al. [34] found that when electrochemically active bacteria adhere to the surface of the anode and form a thick biofilm, the substrate consumption rate increases, thereby increasing the power generation. Makhtar et al. [35] also confirmed that the thickening of the biofilm formed on the surface of the electrode was conducive to the reduction of the internal resistance of the MFC. It could be inferred that the increase in power generation of the mixed cultures MFC was due to the electrochemically active biofilm formed on the electrode surface, which reduced the internal resistance of the MFC.

**Figure 6.**
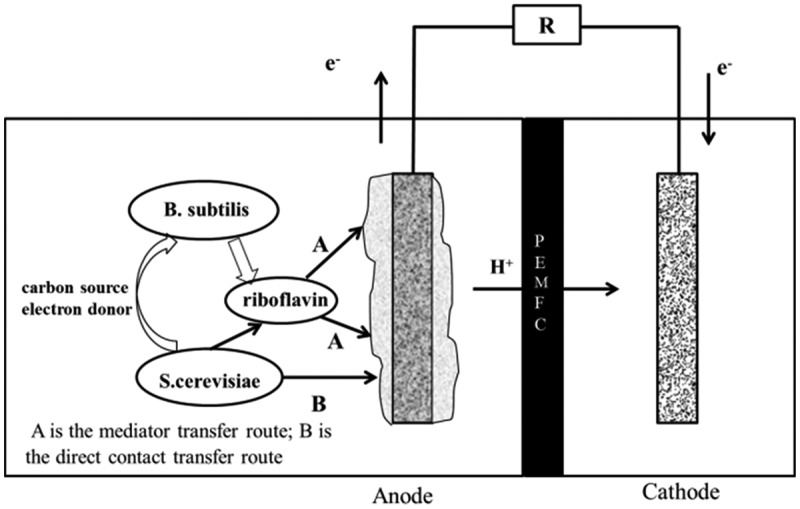
Schematic diagram of the anode electron transfer pathway of mixed cultures MFC

According to the experimental data of this study, we presumed that the mixed bacteria in MFC might have a synergy effect, while the anodic electron transfer mechanism should be: firstly, *Bacillus subtilis* produced electron mediators through respiration to promote the transfer of electrons in the anode and reduced the internal resistance of the MFC. Then, *Saccharomyces cerevisiae* could transfer electrons through the electron mediator produced by *Bacillus subtilis* as well as a direct contact mechanism. Finally, the lactic acid produced by the decomposition of glucose by *Saccharomyces cerevisiae* through respiration served as a carbon source and electron donor for *Bacillus subtilis*. The synergy mechanism is shown in Figure 6.

### 3.3. Synergy effect analysis

To further verified whether the riboflavin synergizing between *Bacillus subtilis* and *Saccharomyces cerevisiae* could be substituted by exogenous riboflavin, 0, 50, 150, and 300 μmol/L riboflavin were added to *Saccharomyces cerevisiae* MFC, *Bacillus subtilis* MFC and mixed cultures MFC with the above two, respectively. Their respective output voltages, and OD_600_ of *Bacillus subtilis* were measured every 24 h.

#### 3.3.1. *Effect of riboflavin concentration on* Saccharomyces cerevisiae *MFC*

**Figure 7.**
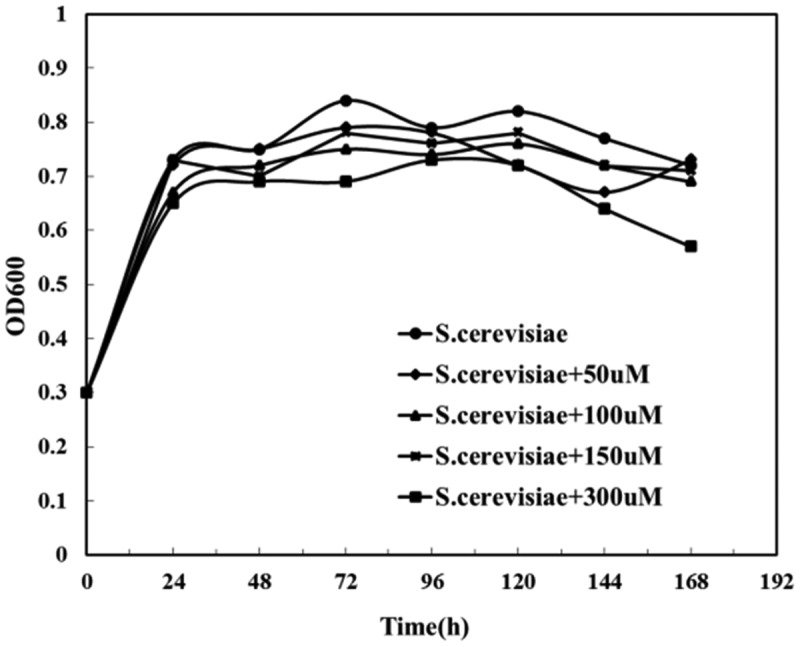
Changes of OD_600_ of *Saccharomyces cerevisiae* under different riboflavin concentrations

When the value of OD_600_ ranges from 0.6 to 0.8, it means that the microorganisms are thriving in the logarithmic phase, while OD_600_ value >3 means that the microorganisms in the culture medium are already saturated, and OD_600_ = 1 means microorganisms are in good condition [36]. As we could see from Figure 7, the OD_600_ of *Saccharomyces cerevisiae* under different riboflavin concentrations showed no obvious changes, suggesting increased riboflavin concentrations made little difference in the respiration of *Saccharomyces cerevisiae*. Therefore, higher concentrations of riboflavin did not help in a bigger number of *Saccharomyces cerevisiae* cells.

**Figure 8.**
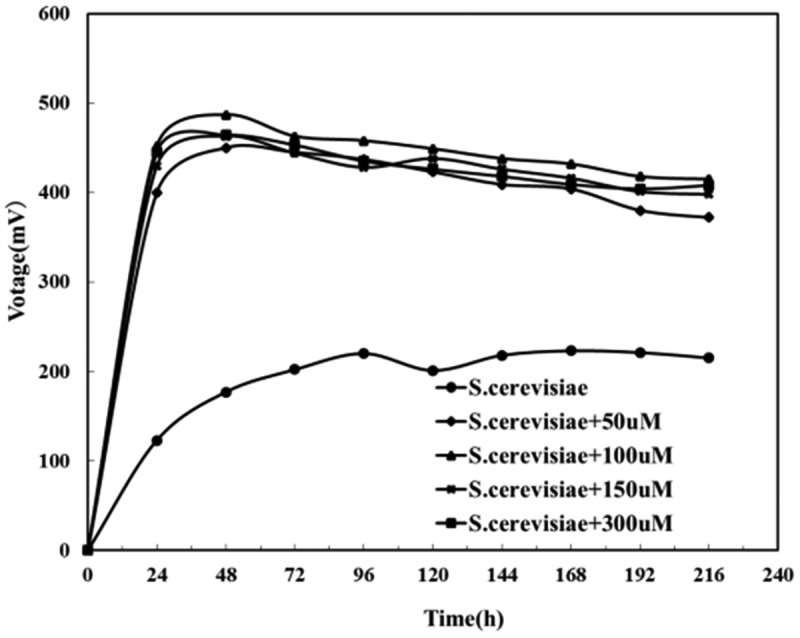
The effect of adding riboflavin on the output voltage of *Saccharomyces cerevisiae.*

From Figure 8, it could be seen that the exogenous riboflavin increases the output voltage of *the Saccharomyces cerevisiae* MFC system, up to 487 mV, which is 220% higher than that in the blank test (220 mV). This indicated that riboflavin, a redox material could be used for *Saccharomyces cerevisiae* to reduce the resistance of its electron transfer and increase the output voltage of the MFC.

#### 3.3.2. Effect of riboflavin concentration on Bacillus subtilis MFC

**Figure 9.**
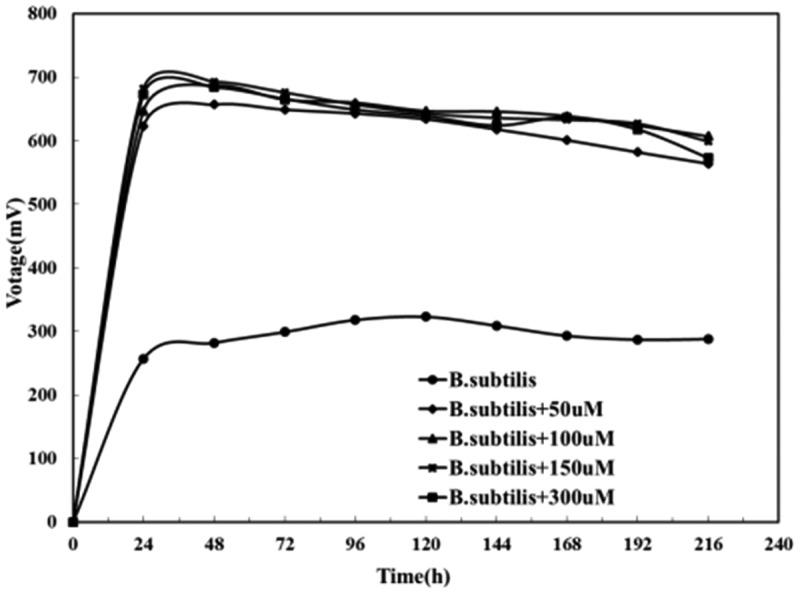
Effects of different riboflavin concentrations on the output voltage of *Bacillus subtilis.*

Figure 9 showed that the output voltage of the *Bacillus subtilis* MFC was 323 mV when riboflavin was absent, while the value increases by 150% (693 mV) with exogenous riboflavin added. It could be concluded that exogenous riboflavin can increase the power generation of the MFC as it created more mediators in the MFC system which could speed up electron transfer.

#### 3.3.3. Effect of riboflavin concentrations on mixed cultures MFC

**Figure 10.**
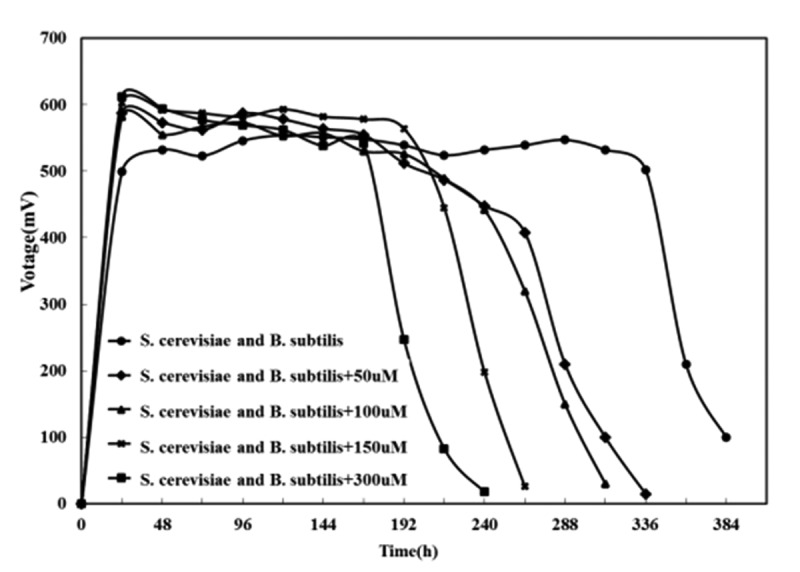
The effect of adding riboflavin on the output voltage of composite bacteria MFC

It was known from the above experiments that the addition of exogenous riboflavin could increase the output voltage of the pure bacterial MFC of *Bacillus subtilis* and *Saccharomyces cerevisiae*, and reduced the internal resistance of the battery. However, it could be seen from Figure 10 that after adding exogenous riboflavin to the mixed culture MFC, the voltage dropped rapidly after being stabilized. Moreover, the higher the concentration of exogenous riboflavin was, the faster the voltage decreased. It was indicated to choose the self-secreting riboflavin over the exogenous riboflavin played the role of electron transfer in this mixed bacterial system, and the over-saturated riboflavin will affect the effect of MFC on electricity generation.

## 4. Conclusions

To overcome the issue of low power yield, the experiments focusing on the synergy effect between electrochemically active bacteria were established, MFC of *Bacillus subtilis* had the best performance on outputed voltage (299 mV). In the mixed culture systems, only MFC composed of *Saccharomyces cerevisiae* and *Bacillus subtilis* had a significant increase in power generation capacity (554 mV) because of the synergy effect. The riboflavin produced by *Bacillus subtilis* was utilized by *Saccharomyces cerevisiae* for electron transfer, and S*accharomyces cerevisiae* provided carbon source and electron donor for *Bacillus subtilis*. The excess riboflavin might hinder electron transfer in mixed cultures MFC.

## Data Availability

The data used to support the findings of this study are available from the corresponding author upon request.

## Research highlights

- Explored the synergistic effect of mixed cultures MFC
- Analyzed the potential and feasibility of MFC based mixed technologies
- The synergistic effect of *S. cerevisiae* and *B. subtilis* improved the power generation

**Theme words**:

Bioenergy and Biofuels

Bioremediation, biodegradation, and bioaccumulation

Microbial Biotechnology

## References

[1] Choudhury P, Uday U, Bandyopdhyay T, et al. Performance improvement of microbial fuel cell (MFC) using suitable electrode and Bioengineered organisms: A review. Bioengineered. 2017;8(5):471–487. 28453385 10.1080/21655979.2016.1267883 PMC5639845

[2] Chaturvedi V, Verma P. Microbial fuel cell: a green approach for the utilization of waste for the generation of bioelectricity. Bioresources Bioprocess. 2016;3(1):1–14.

[3] Liu J, Zhao N, Ge L. Research progress on electron transfer mechanism and its influencing factors on microbial fuel cells anode exoelectrogens. Environ Chem. 2019;38(8):1–12.

[4] Dao-Bo L, Edwards Marcus J, Blake Anthony W, et al. His/Met heme ligation in the PioA outer membrane cytochrome enabling light-driven extracellular electron transfer by Rhodopseudomonas palustris TIE-1. Nanotechnology. 2020;31(35):354002. 32403091 10.1088/1361-6528/ab92c7

[5] Zhao Y, Watanabe K Prof, Nakamura R, et al. Three-dimensional conductive nanowire networks for maximizing anode performance in microbial fuel cells. Chem A Eur J. 2010;16(17):4982–4985. 10.1002/chem.200903486 20340117

[6] Kartik A, Vijayakumar BS. Screening sediment samples used as anolytes in microbial fuel cells for microbial electron transfer activity using DREAM assay. Biotechnol Lett. 2019;41(8–9):979–985. 31236788 10.1007/s10529-019-02704-3

[7] Song R, Wu Y, Lin Z. Coating individual bacterial cells with in situ formed polypyrrole. Living Cond. 2017;56(35):10516–10520. 10.1002/anie.201704729 28590548

[8] Ni J, Chen X, Wang X, et al. Research progress of electrogenic microorganisms in microbial fuel cell. Modern Chem Ind. 2017;37(7):46–49.

[9] Reguera G, Nevin KP, Nicoll JS. Biofilm and nanowire production leads to increased current in Geobacter sulfurreducens fuel cells. Appl Environ Microbiol. 2006;72(11):7345–7348. 16936064 10.1128/AEM.01444-06 PMC1636155

[10] Mohammadi Moradian J, Xu Z-A, Shi Y-T, et al. Efficient biohydrogen and bioelectricity production from xylose by microbial fuel cell with newly isolated yeast of Cystobasidium slooffiae. Int J Energy Res. 2020;44(1):325–333.

[11] Rabaey K, Boon N, Hofte M. Microbial phenazine production enhances electron transfer in biofuel cells. Environ Sci Technol. 2005;39(9):3401–3408. 15926596 10.1021/es048563o

[12] Fathey R, Gomaa M, Ali E. Neutral red as a mediator for the enhancement of electricity production using a domestic wastewater double chamber microbial fuel cell. Ann Microbiol. 2016;66(2):695–702.

[13] Tingtao Z, Lixia Z, Ping G. The mechanism and characteristics of electricity production in compound strains microbial fuel cells. J App Environ Biol. 2012;18(3):465–470.

[14] Rui L, Tursun H, Xiaoshu H, et al. Microbial community dynamics in a pilot-scale MFC-AA/O system treating domestic sewage. Bioresour Technol. 2017;241:439–447. 28599222 10.1016/j.biortech.2017.05.122

[15] Park DH, Zeukus JG. Impact of electrode composition on electricity generation in a single compartment fuel cell using Shewanella putrefaciens. Appl Microbiol Biotechnol. 2002;59:58–61. 10.1007/s00253-002-0972-1 12073132

[16] Raghavulu SV, Modestra JA, Amulya K, et al. Relative effect of bioaugmentation with electrochemically active and nonactive bacteria on bioelectrogenesis in microbial fuel cell. Bioresour Technol. 2013;146:696–703. 23988904 10.1016/j.biortech.2013.07.097

[17] Yujin C, Hui M, Wei L. Electricigens in the anode of microbial fuel cells: pure cultures versus mixed communities. Microb Cell Fact. 2019;18:18–39. 30782155 10.1186/s12934-019-1087-z PMC6380051

[18] Islam MA, Ethiraj B, Cheng CK, et al. An insight of synergy between Pseudomonas aeruginosa and Klebsiella variicola in microbial fuel cell. ACS Sustain Chem Eng. 2018;6:4130-4137.

[19] Islam MA, Woon CW, Ethiraj B, et al. Correlation of power generation with time-course biofilm architecture using Klebsiella variicola in dual chamber microbial fuel cell. Int J Hydrogen Energy. 2017;42:25933–25941.

[20] Kimberley D, Duarte Z, Kwon Y. In situ carbon felt anode modification via codeveloping Saccharomyces cerevisiae living-template titanium dioxide nanoclusters in a yeast-based microbial fuel cell. J Power Sources. 2020;228651:474.

[21] Sugnaux M, Mermoud S, Costa AFD. Probing electron transfer with Escherichia coli: A method to examine exoelectronics in microbial fuel cell type systems. Bioresour Technol. 2013;148(8):567–573. 24080296 10.1016/j.biortech.2013.09.004

[22] Qiao Y, Qiao YJ, Zou L. Real-time monitoring of phenazines excretion in Pseudomonas aeruginosa microbial fuel cell anode using cavity microelectrodes. Bioresour Technol. 2015;198:1–6. 26360598 10.1016/j.biortech.2015.09.002

[23] Feng L, Xingjuan A, Deguang W. Engineering microbial consortia for high-performance cellulosic hydrolyzates-fed microbial fuel cells. Front Microbiol. 2019;10:409. 30936852 10.3389/fmicb.2019.00409 PMC6432859

[24] Kokabian B, Smith R, Brooks JP. Bioelectricity production in photosynthetic microbial desalination cells under different flow configurations. J Ind Eng Chem. 2018;58:131–139.

[25] Raghavulu SV, Goud RK, Sarma PN, et al. Saccharomyces cerevisiae as anodic biocatalyst for power generation in biofuel cell: influence of redox condition and substrate load. Bioresour Technol. 2011;102:2751–2757. 21146401 10.1016/j.biortech.2010.11.048

[26] Ojima, Y, Kawaguchi T, Fukui S, et al. Promoted performance of microbial fuel cells using Escherichia coli cells with multiple-knockout of central metabolism genes. Bioprocess Biosyst Eng. 2019;43(2):323–332. 31606755 10.1007/s00449-019-02229-z

[27] Qian Z, YANG L, XIE B. Isolation and identification of a simultaneous electricity production and denitrification strain in a microbial fuel cell with biocathode and its characteristics. Chin J Environ Eng. 2019;13(8):1986–1994.

[28] Nimje VR, Chen CY, Chen CC, et al. Stable and high energy generation by a strain of Bacillus subtilis in a microbial fuel cell. J Power Sources. 2009;190(2):258–263.

[29] You T, Liu JH, Liang RB, et al. Longer survival time of Pseudomonas aeruginosa could increase electricity production of biofuel cells. J Bioeng. 2017;33(4):601–608. 10.13345/j.cjb.160372 28920393

[30] Rossi. R, Cavina M, Setti L. Characterization of electron transfer mechanism in mediated microbial fuel cell by entrapped electron mediator in Saccharomyces cerevisiae. Chem Eng Trans. 2016;49.

[31] Olja S, Marta S, Eberlin Livia S, et al. Electrochemical monitoring of the impact of polymicrobial infections on pseudomonas aeruginosa and growth dependent medium. Biosens Bioelectron. 2019;142:111538. 31376710 10.1016/j.bios.2019.111538

[32] Moradian JM, Xu Z, Shi Y. Efficient biohydrogen and bioelectricity production from xylose by microbial fuel cell with newly isolated yeast of Cystobasidium slooffiae. Int J Energy Res. 2020;44(1):325–333.

[33] Wu S, Xiao Y, Song P. Riboflavin-mediated extracellular electron transfer process involving Pachysolen tannophilus. Electrochim Acta. 2016;210:117–121.

[34] Zhi W, Ge Z, He Z, et al. Methods for understanding microbial community structures and functions in microbial fuel cells: a review. Bioresour Technol. 2014;171:461–468. 25223851 10.1016/j.biortech.2014.08.096

[35] Makhtar MMZ, Vadivelu VM. Membraneless microbial fuel cell: characterization of electrogenic bacteria and kinetic growth model. J Environ Eng. 2019;145(5):1-7.

[36] Ze Chao X, Yuan Yuan C, Wan Qin K, et al. Electron shuttles alter selenite reduction pathway and redistribute formed Se(0) nanoparticles. Process Biochem. 2016;51(3):408–413.

